# A Successful Treatment of Endoscopic Third Ventriculostomy with Choroid Plexus Cauterization for Hydrocephalus in Walker-Warburg Syndrome

**DOI:** 10.1155/2016/7627289

**Published:** 2016-12-27

**Authors:** Tomoko Tanaka, Catharine J. Harris, Sarah S. Barnett, N. Scott Litofsky

**Affiliations:** ^1^Division of Neurosurgery, University of Missouri, One Hospital Drive, Columbia, MO 65212, USA; ^2^Division of Genetics, University of Missouri, One Hospital Drive, Columbia, MO 65212, USA

## Abstract

Walker-Warburg syndrome (WWS) is a rare autosomal recessive congenital muscular dystrophy with brain malformations and ocular abnormalities that falls under the wider phenotypic spectrum of the dystroglycanopathies. Mutations in a number of genes including POMT1, POMT2, POMGNT1, POMGNT2, FKTN, FKRP, LARGE, and ISPD are known to cause alpha dystroglycan-related muscular dystrophy. Mutations in these genes result in a broad phenotypic spectrum ranging from the severe WWS to a mild congenital muscular dystrophy with no brain involvement. WWS is fatal to most patients early in life with mean survival of 9 months. The most common brain finding is cobblestone lissencephaly with the vast majority of patients (97%) also having ventricular dilation with or without hydrocephalus. Surgical treatment has not been frequently detailed. This report describes our successful treatment of a patient with WWS and hydrocephalus with Endoscopic Third Ventriculostomy (ETV) with choroid plexus cauterization (CPC). Fourteen months following treatment, a follow-up MRI CSF flow study demonstrated robust CSF flow through floor of third ventricle from interpeduncular cistern to lateral ventricle.

## 1. Introduction

Walker-Warburg syndrome (WWS) is a rare autosomal recessive congenital muscular dystrophy with brain malformations and ocular abnormalities and a prevalence of 1 in 100,000 births. It is a fatal condition with average life expectancy usually less than 3 years. Hydrocephalus is a known concomitant condition. In patients with hydrocephalus from other causes, choroid plexus cauterization (CPC) has been associated with improvement of Endoscopic Third Ventriculostomy (ETV) outcome compared to ETV alone. Utilizing combination of ETV and CPC treatment, we demonstrated a successful outcome in a WWS patient. Despite a low predictive ETV success score, patients with WWS and concomitant hydrocephalus can be successfully treated with ETV and CPC, improving their quality of life over their limited lifespan, as this case illustrates.

## 2. Case Presentation

### 2.1. History and Examination

The patient was a female infant delivered by cesarean section at 37 weeks 2 days gestation age due to dichorionic/diamniotic twin pregnancy. Apgar scores were 6 and 9 and birth weight was 2021 g. The patient was noted to be small for gestational age, hypothermia, and hypotonic. She was admitted to the neonatal intensive care unit. A prenatal ultrasound at 20 weeks revealed enlarged ventricles and enlarged posterior fossa fluid spaces, while a postnatal brain MRI demonstrated a delayed myelination pattern, enlarged gyri, cerebellar hypoplasia, and enlarged ventricles including lateral, third, and fourth; findings were consistent with Dandy-Walker Malformation. At that time, no clinical signs suggested increased intracranial pressure; the anterior fontanel was soft and sutures were opposed. Therefore, treatment of enlargement of the ventricles was deferred. A G-tube was placed at three weeks of age for failure to thrive, and the patient was discharged home. Her twin brother had no medical problems.

**Figure 1:**
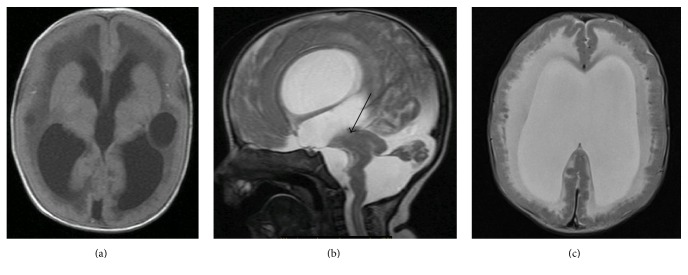
(a) Axial FLAIR: agyria enlarged ventricle, bilateral periventricle cystic hypodenisty, and delayed myelination of cerebral cortex. (b) Sagittal T2: enlarged lateral and third ventricle. Hypoplastic cerebellum, large posterior fossa CSF fluid space. There is no visible aqueduct (arrow), kinked pontomesencephalic kink. (c) Axial T2-weighted image reveals typical changes related to cobblestone lissencephaly. White matter is diffusely hyperintensity. Enlarged ventricle.

Genetic consultation was obtained as an outpatient; due to muscle, eye, and brain abnormalities the patient was suspected to have Walker-Warburg syndrome. Diagnostic testing revealed normal TORCH titers, normal chromosomal microarray, normal 7-dehydrocholesterol and compound heterozygous mutations in POMT1 consistent with WWS [c.1153C>T and c.2167dupG]. An ophthalmology examination confirmed bilateral glaucoma, cataract, optic nerve hypoplasia, and chorioretinal atrophy. At routine neurosurgical follow-up around 4.5 months of age, the parents reported increasing irritability. Repeat MRI did not show significant difference in ventricle size compared to the MRI at birth. However, it demonstrated significant findings of delayed myelination of cerebral cortex, hypoplastic cerebellum, and cobblestone lissencephaly (Figures 1(a)–1(c)), all of which are well-characterized in WWS. Clinical signs of increasing intracranial pressure with bulging anterior fontanelle and sutural separation were present as well. The patient also demonstrated increased seizure activity. Due to the suspicion of aqueduct stenosis and concerns about the patient's baseline elevation of CPK level (5,055 unit/L), the decision was made to treat with an ETV and CPC over placement of a ventriculoperitoneal shunt.

### 2.2. Operation

**Figure 2:**
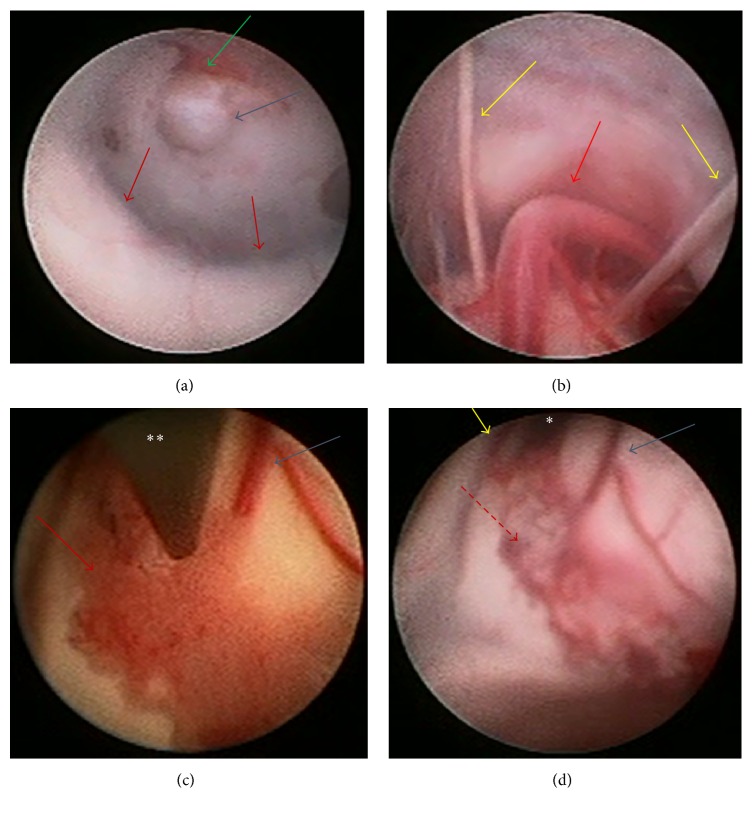
(a) Ventriculostomy was fenestrated at infundibulum recess. Blue arrow: ventriculostomy, green arrow: clivus, and red arrows: bilateral mammillary body. (b) Endoscopic view through ventriculostomy in the interpeduncular cistern. Red arrow: basilar artery and yellow arrows: bilateral oculomotor nerves.  ^*∗*^Foramen of Monro.  ^*∗∗*^Endoscope.

Following intubation and pharmacological induction, an Aesculap PaediScope® Endoscope was inserted in right lateral ventricle. Anatomical distortion was seen during the endoscopic examination of the ventricle (Figure 2(a)). There was no septum pellucidem, and a periventricular cyst was seen, consistent with the MRI findings. Mammillary bodies were slightly stretched laterally, and the infundibulum recess and dorsum sella were well visualized. The third ventriculostomy was performed just behind the dorsum sella. Success of ETV was confirmed as the endoscope was advanced into the prepontine cistern to observe the basilar artery and oculomotor cranial nerves (Figure 2(b)). Following completion of the third ventriculostomy, endoscopic monopolar cautery was applied to dorsal portion of choroid plexus from foramen of Monro to trigone of the right lateral ventricle without injury to the septal vein, the thalamostriate vein, or the thalamus. No significant bleeding occurred. Only about 10% of the choroid plexus remained on the right. The choroid plexus on the left was not coagulated because the flexible endoscope was not available to coagulate contralateral side. Since the third ventriculostomy site demonstrated vigorous pulsation (Figures 2(c) and 2(d)), additional risk was not warranted. The consideration was to avoid prolonging operating time, which could contribute to increasing CPK. We felt the intervention accomplished would be sufficient.

### 2.3. Postoperative Course

**Figure 3:**
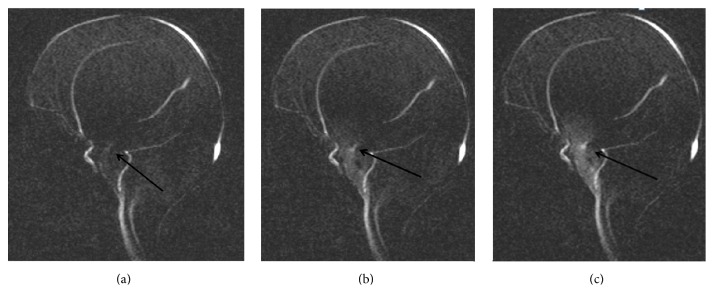
(a, b, c) MRI CSF flow study: arrow shows CSF flow at the floor of third ventricle.

**Figure 4:**
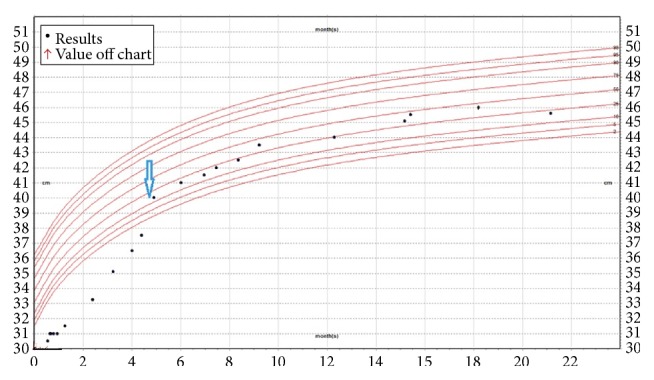
Patient occipital-frontal circumference chart. Blue arrow showed time of ETV with CPC.

The patient tolerated the procedure well and went home the following day. Her head circumference stabilized, sutures became opposed and the anterior fontanelle softened. Postoperatively, her irritability has improved dramatically and her seizures were controlled well. At 15 months after surgery, a follow-up brain MRI with flow study showed robust CSF pulsation at floor of third ventricle (Figures 3(a), 3(b), and 3(c)). The patient's head circumference followed a stable growth curve (Figure 4). She remained globally delayed, and never rolled over or sat by herself. At 21 months of age she was found deceased at home. A specific cause of death was not determined as she did not have an autopsy.

## 3. Discussion

Walker-Warburg syndrome (WWS) is a rare autosomal recessive disorder with a prevalence of 1 in 100,000 births [1]. Clinical characteristics include a congenital muscular dystrophy, brain malformations, and ocular abnormalities. The syndrome is caused by defects in O-linked protein glycosylation. Walker first described the syndrome in 1942, noting agyria, hydrocephalus, and eye malformations. Warburg subsequently suggested a distinctive syndrome with hydrocephaly, congenital retinal detachment, and congenital facial folds in 1978 [2]. The diagnostic criteria for WWS were established by Dobyns et al. in 1989 [3]. Mutations in several genes are currently known to lead to a muscular dystrophy-dystroglycanopathy including POMT1, POMT2, POMGNT1, FKTN, FKRP, LARGE, GTDC2, G3GALNT2, GMPPB, B3GNT1, TMEM5, COL4A1, and ISPD. Even so, the genetic etiology remains undefined in about 30% of cases [4]. Due to the autosomal recessive inheritance pattern, reports of siblings with WWS occur; however our patient's twin brother was unaffected [5, 6].

Failure of the glycosylation process leads to characteristic MRI findings which include type II (“cobblestone”) lissencephaly and myelination failure. Other findings include a nearly agyric cerebrum, polymicrogyria of the cerebellar cortex, cysts, enlarged lateral ventricles, agenesis of the corpus callosum, pontine hypoplasia, and a kinked pontomesencephalic junction [7, 8]. Life expectancy is significantly reduced, usually less than 3 years, with an average of 9 months [3].

When symptomatic ventriculomegaly or hydrocephalus is identified, treatment should be seriously considered. Untreated hydrocephalus with increased intracranial pressure may lead to cause of death or shorten patient lifespan. Due to the severe nature of the disease, parents may elect against surgery and the patient may compensate for the hydrocephalus for a period of time without surgical intervention. However, macrocephaly can lead to mechanical instability of cortical mantel, failure of head control, problems with head positioning, skin breakdown, and other difficulties [9]. More importantly, the irritability associated with untreated hydrocephalus is uncomfortable and worrisome for both the patient and the family, as reported by the parents in this case.

We successfully treated hydrocephalus in our patient with WWS by ETV with choroid plexus cauterization (CPC). CPC has been described as a useful adjunct to ETV [10] (Table 1). Based on the data of Klukarini [11], we predicted a suboptimal success rate in our patient of 40%. Therefore, CPC was performed in addition to ETV to improve the patient's long term outcome. Warf et al. reported success rate of 48.6% for congenital idiopathic hydrocephalus treatment in Uganda with ETV alone compared a success rate of ETV with CPC of 81.9% [12]. In their meta-analysis, Zandian et al. concluded that the success rate of ETV with CPC was 67% compared to 55% for ETV alone [13]. Clearly, the addition of CPC provides value added to ETV, particularly in patients with low predictive ETV success score [11].

Our patient had WWS with severe congenital disease. We had high suspicion of aqueduct stenosis on the MRI study, supporting our plan to achieve success utilizing ETV along with CPC. Preuss et al. previously reported a single case of hydrocephalus in WWS treated by ETV without CPC [14]. In their case, long term success was not achieved and the patient required placement of ventricle-peritoneal shunt 4 months later. Preuss et al. concluded that early failure of ETV occurred because their patient had abnormal anatomical landmarks which documented absence of mamillary bodies, small thalamus, agenesis of anterior and posterior commissure, and complete atresia of the Sylvian aqueduct. Therefore, ETV was performed rostral to lamina terminalis. Although our patient had some anatomic distortions, the location of the mammillary bodies was such that we could position our ETV just posterior to the dorsum sella. Knowing that our patient had a suboptimal predictive ETV success score (40) (Table 1) [11], the addition of choroid plexus cauterization was discussed with the parents. We outlined our goal of treatment for palliative CSF diversion with CPC to optimize long term success of ETV. Increased CPK has been observed after neurosurgical procedures, although it typically reported with longer craniotomy and spine procedures [15]. While a ventriculoperitoneal shunt could have been placed instead, the already high CPK level due to the muscle dystrophy seen in WWS patients was of concern, as this level could further increase with passing the shunt passer and contribute to renal insufficiency. These concerns were the subject of extensive discussion with her parents prior to the procedure. Though the family was initially not interested in surgery of any kind, the patient's increasing irritability and discomfort led the parents to elect for surgical intervention.

The reason of unsuccessful outcome of previous reported case [14] was unclear. The authors concluded that anatomical distortion might be contributed failure of ETV. The predictive ETV success score in the previous case was 30 compared to our patient of 40 since their patient was less than 1 month old. Failure in children less than 3 months old was previously observed by Chamiraju et al. [16]. In their series, they identified higher surgical success outcome of ETV with CPC if age exceeded 3 months (50% versus 18.2%), weight was more than 3 kg (47% versus 20%), and anatomical findings in the prepontine cistern were normal compared to scarring (47.5% versus 12.5%) in posthemorrhagic hydrocephalus in premature infants [16]. Therefore, the combination of low ETV success score and anatomical abnormality may have affected previously reported case. Perhaps CPC would have contributed to better success in that patient.

## 4. Conclusions

Despite a low predicted ETV success score, patients with WWS and concomitant hydrocephalus can be successfully treated with ETV and CPC, improving their quality of life over their limited lifespan, as this case illustrates.

## Figures and Tables

**Table 1:** Kulkarni's ETV success core [11].

Calculation of the ETVSS^*∗*^			
Score	Age	Etiology	Previous Shunt
0	<1 mo	Postinfectious	Previous shunt
10	1 mo to <6 mos		No previous shunt
20		Myelomeningocele, IVH, nontectal brain tumor	
30	6 mos to <1 yr	Aqueductal stenosis, tectal tumor, other	
40	1 yr to <10 yrs		
50	≥10 yrs		

^*∗*^The ETVSS is calculated as age score + etiology score + previous shunt score. IVH = intraventricular hemorrhage.
